# *CRLF2* over-expression is a poor prognostic marker in children with high risk T-cell acute lymphoblastic leukemia

**DOI:** 10.18632/oncotarget.10610

**Published:** 2016-07-15

**Authors:** Chiara Palmi, Angela M. Savino, Daniela Silvestri, Ilaria Bronzini, Gunnar Cario, Maddalena Paganin, Barbara Buldini, Marta Galbiati, Martina U. Muckenthaler, Cristina Bugarin, Pamela Della Mina, Stefan Nagel, Elena Barisone, Fiorina Casale, Franco Locatelli, Luca Lo Nigro, Concetta Micalizzi, Rosanna Parasole, Andrea Pession, Maria C. Putti, Nicola Santoro, Anna M. Testi, Ottavio Ziino, Andreas E. Kulozik, Martin Zimmermann, Martin Schrappe, Antonello Villa, Giuseppe Gaipa, Giuseppe Basso, Andrea Biondi, Maria G. Valsecchi, Martin Stanulla, Valentino Conter, Geertruy te Kronnie, Giovanni Cazzaniga

**Affiliations:** ^1^ Centro Ricerca M. Tettamanti, Clinica Pediatrica, Università di Milano Bicocca, Fondazione MBBM/Ospedale San Gerardo, Monza, Italy; ^2^ Center of Biostatistics for Clinical Epidemiology, Department of Health Sciences, University of Milano-Bicocca, Milan, Italy; ^3^ Clinica Pediatrica, Università di Milano Bicocca, Fondazione MBBM/Ospedale San Gerardo, Monza, Italy; ^4^ Laboratory of Onco-Hematology, Department SDB, Università di Padova, Padova, Italy; ^5^ Department of Pediatrics, University Hospital Schleswig-Holstein, Campus Kiel, Kiel, Germany; ^6^ Department of Pediatric Oncology, Hematology and Immunology, University of Heidelberg and EMBL/Medical Faculty Molecular Medicine Partnership Unit, Heidelberg, Germany; ^7^ Microscopy and Image Analysis Consortium, Università di Milano-Bicocca, Monza, Italy; ^8^ Department of Human and Animal Cell Lines, Leibniz-Institute DSMZ - German Collection of Microorganisms and Cell Cultures, Braunschweig, Germany; ^9^ Pediatric Onco-Hematology, Stem Cell Transplantation and Cellular Therapy Division, Regina Margherita Children's Hospital, Turin, Italy; ^10^ Pediatric Oncology Service, Pediatric Department of 2nd University of Naples, Naples, Italy; ^11^ Department of Pediatric Hematology/Oncology, IRCCS Ospedale Bambino Gesù, Rome - University of Pavia, Pavia, Italy; ^12^ Center of Pediatric Hematology Oncology, Azienda Ospedaliero-Universitaria “Policlinico Vittorio Emanuele”, Catania, Italy; ^13^ Hematology/Oncology Unit, G. Gaslini Children's Hospital, Genoa, Italy; ^14^ Department of Pediatric Hemato-Oncology, Ospedale Pausilipon, Napoli, Italy; ^15^ Department of Pediatrics, “Lalla Seràgnoli” Hematology-Oncology Unit, University of Bologna, Bologna, Italy; ^16^ Department of Pediatrics, Division of Pediatric Hematology-Oncology, University “A. Moro” of Bari, Bari, Italy; ^17^ Division of Hematology, Department of Biotechnologies and Hematology, “Sapienza” University of Rome, Rome, Italy; ^18^ Pediatric Hematology and Oncology Unit, A.R.N.A.S. Civico, Di Cristina and Benfratelli Hospital, Palermo, Italy; ^19^ Department of Paediatric Haematology and Oncology, Hannover Medical School, Hannover, Germany

**Keywords:** CRLF2, pediatric leukemia, T acute lymphoblastic leukemia, prognostic marker, high risk

## Abstract

Pediatric T-ALL patients have a worse outcome compared to BCP-ALL patients and they could benefit from new prognostic marker identification. Alteration of *CRLF2* gene, a hallmark correlated with poor outcome in BCP-ALL, has not been reported in T-ALL.

We analyzed *CRLF2* expression in 212 T-ALL pediatric patients enrolled in AIEOP-BFM ALL2000 study in Italian and German centers.

Seventeen out of 120 (14.2%) Italian patients presented *CRLF2* mRNA expression 5 times higher than the median (*CRLF2-high*); they had a significantly inferior event-free survival (41.2%±11.9 vs. 68.9%±4.6, p=0.006) and overall survival (47.1%±12.1 vs. 73.8%±4.3, p=0.009) and an increased cumulative incidence of relapse/resistance (52.9%±12.1 vs. 26.2%±4.3, p=0.007) compared to *CRLF2-low* patients. The prognostic value of *CRLF2* over-expression was validated in the German cohort. Of note, *CRLF2* over-expression was associated with poor prognosis in the high risk (HR) subgroup where *CRLF2-high* patients were more frequently allocated.

Interestingly, although in T-ALL CRLF2 protein was localized mainly in the cytoplasm, in *CRLF2-high* blasts we found a trend towards a stronger TSLP-induced pSTAT5 response, sensitive to the JAK inhibitor Ruxolitinib.

In conclusion, *CRLF2* over-expression is a poor prognostic marker identifying a subset of HR T-ALL patients that could benefit from alternative therapy, potentially targeting the *CRLF2* pathway.

## INTRODUCTION

Notwithstanding improved survival rates obtained with risk-adjusted therapy, 25% of T-ALL patients have little or no expectancy of cure. Indeed, this ALL subtype has a generally worse outcome compared with BCP-ALL [1, 2] and the prognosis after relapse remains dismal [3]. In the AIEOP-BFM ALL 2000 study, risk group stratification was largely based on Minimal Residual Disease (MRD) monitoring as a measure of early response to therapy [1, 2]. In BCP-ALL, chromosomal translocations have been also incorporated in the risk stratification employed for choosing treatment [4, 5]. By contrast in T-ALL, although several genomic abnormalities have been described, only few shown to have prognostic value, and none has been included in treatment protocols as criteria for patient stratification [6–13]. Hence, identification of prognostic factors and development of innovative therapeutic approaches for T-ALL remain a critical task for leukemia research.

Among recently reported genomic abnormalities in ALL, a subset of BCP-ALL patients has been characterized by over-expression of the Cytokine Receptor-like Factor 2 (*CRLF2*) gene, associated with either an intra-chromosomal deletion causing the *P2RY8*-*CRLF2* fusion or the *IGH@-CRLF2* translocation [14, 15]. These two *CRLF2* rearrangements have been shown to correlate with poor outcome in BCP-ALL patients [16–20].

CRLF2 heterodimerizes with IL-7Rα to form a receptor for thymic stromal lymphopoietin (TSLP), an epithelial cell-derived cytokine that regulates dendritic cells (DC)-mediated central tolerance, peripheral T cell homeostasis and inflammatory Th2 responses. [21] Signaling from TSLP receptor activates signal transducer and activator of transcription (STAT5) by JAK1 and JAK2 phosphorylation [22, 23].

*CRLF2* rearrangements are a new prognostic marker for BCP-ALL, and the inhibition of JAK/STAT5 signaling represents a potential new therapeutic approach for this subgroup of patients.

Alterations of *CRLF2* have not yet been reported in T-ALL, while recently mutations in its partner *IL7Rα* have been identified in about 10% of T-ALL patients [24, 25]. This observation prompted us to investigate if CRLF2 could also be affected in T-ALL.

Here, we report on the incidence and prognostic impact of *CRLF2* over-expression at diagnosis in 212 T-ALL patients, enrolled in Italian and German centers in the protocol of the Associazione Italiana Ematologia Oncologia Pediatrica (AIEOP) and the Berlin-Frankfurt-Munster (BFM) groups (AIEOP-BFM ALL 2000 protocol).

## RESULTS

### *CRLF2* alterations and other genetic aberrations in AIEOP T-ALL patients at diagnosis

**Figure 1:**
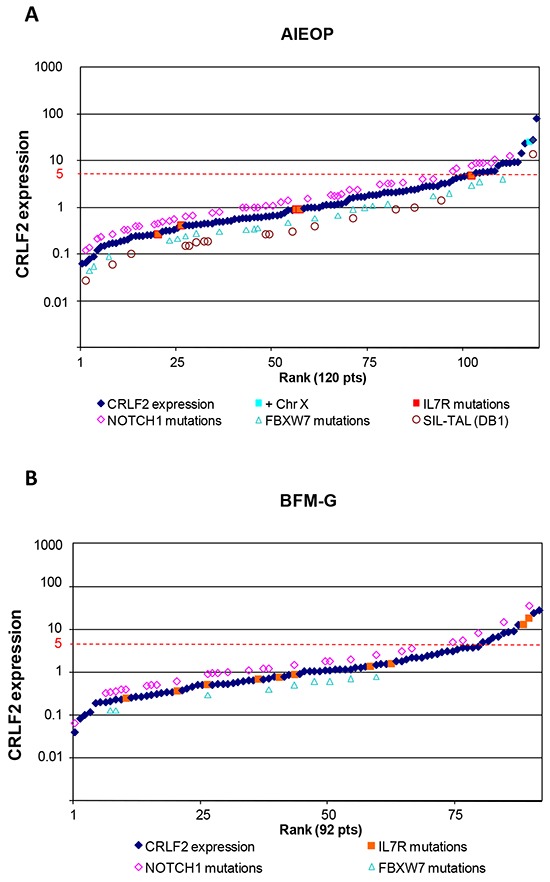
*CRLF2* expression and genomic alterations *CRLF2* expression in AIEOP **A.** and BFM-G **B.** T-ALL patients at diagnosis. For each specimen, results are reported as fold changes on the median expression value of their respective cohort. Positivity for additional genomic aberrations is indicated.

Similarly to what is seen in BCP-ALL, [16, 19] a sigmoid curve was observed for the distribution of *CRLF2* expression levels in AIEOP T-ALL patients, with *CRLF2* expression at diagnosis ranging from a 0.06- to an 82- fold change with respect to the median value (Figure 1A). The median delta Ct of the T-ALL cohort was comparable to that of the BCP-ALL (3.36 vs. 3.05, respectively) [19].

As previously reported for *CRLF2* expression in BCP-ALL, [19] in order to define *CRLF2* high-expressing (“*CRLF2-high”*) patients, the CIR hazard ratio was calculated for each unit increase in the *CRLF2* expression expressed as fold change with respect to the median value. The lowest threshold for *CRLF2* expression showing a significant difference (p≤0.01) in CIR was 5 times the median, which was adopted as cut-point. (Supplementary Figure S1).

Seventeen patients out of 120 (14.2%) presented *CRLF2* expression 5 times higher or equal than the median.

Clinical characteristics of *CRLF2-high* patients at diagnosis vs. *CRLF2-low* patients are reported in Table 1. Unlike *CRLF2-low* patients, the majority of *CRLF2-high* patients were poor prednisone responders (PPR) (10/17 patients, 58.8%; p=0.02), while no significant differences were observed with respect to sex, age, WBC count and immunophenotypic subtype (in particular 2 *CRLF2-low* patients vs. 1 *CRLF2-high* fulfilled the immunophenotypic criteria to be classified as early T-cell precursor ALL (ETP-ALL), data not shown). Although *CRLF2* over-expression did not statistically correlate with PCR-MRD classification, consistent with the more frequent incidence of PPR, *CRLF2-high* patients were frequently allocated to the HR group (Table 1). Among *CRLF2-high* cases we verified that *CRLF2* expression levels were similar in cases with high risk features compared to the cases without high risk features (Supplementary Table S1).

**Table 1:** Clinical features of AIEOP and BFM-G study cohort patients positive or negative for *CRLF2* overexpression

Characteristics	AIEOP					BFM-G				
	P-value	*CRLF2-low*		*CRLF2-high*		P-value	*CRLF2-low*		*CRLF2-high*	
		N	%	N	%		N	%	N	%
**All patients**		103	100	17	100		80	100	12	100
**Gender**	0.40					0.99				
** Male**		82	79.6	12	70.6		62	77.5	9	75.0
** Female**		21	20.4	5	29.4		18	22.5	3	25.0
**Age**	0.48					0.15				
** 1-5 Yrs**		38	36.9	5	29.4		23	28.8	1	8.3
** 6-9 Yrs**		22	21.4	4	23.5		24	30.0	3	25.0
** 10-14 Yrs**		36	35.0	5	29.4		24	30.0	4	33.3
** 15-17 Yrs**		7	6.8	3	17.6		9	11.3	4	33.3
**WBC (X1000/ul)**	0.21					0.42				
** <20**		26	25.2	1	5.9		6	7.5	2	16.7
** 20-100**		34	33.0	7	41.2		27	33.8	5	41.7
** ≥ 100**		43	41.7	9	52.9		47	58.8	5	41.7
**Immunophenotype**	0.93					<0.001				
** Early-T**		30	29.1	6	35.3		8	10.0	6	50.0
** Thym**		55	53.4	9	52.9		62	77.5	4	33.3
** Mature T**		13	12.6	2	11.8		9	11.3	2	16.7
** Not specified**		5	4.9	0	0		1	1.3	0	0
**Predn. Response**	0.02					0.09				
** Good**		70	68.0	7	41.2		53	66.3	4	33.3
** Poor**		31	30.1	10	58.8		27	33.8	7	58.3
** Unknown**		2	1.9	0	0		0	0	1	8.3
**MRD**	0.73					0.88				
** SR**		15	14.6	1	5.9		10	12.5	1	8.3
** MR**		35	34.0	5	29.4		51	63.8	5	41.7
** HR**		18	17.5	3	17.6		13	16.3	2	16.7
** Unknown**		35	34.0	8	47.1		6	7.5	4	33.3
**Final Risk**	0.05					0.11				
** no-HR**		62	60.2	6	35.3		49	61.3	4	33.3
** HR**		41	39.8	11	64.7		31	38.8	8	66.7
***P2RY8-CRLF2***	-					-				
** *No***		90	87.4	16	94.1		78	97.5	12	100
** *Yes***		0	0	0	0		0	0	0	0
** *Unknown***		13	12.6	1	5.9		2	2.5	0	0

WBC, White Blood Cell count; MRD, Minimal Residual Disease; HR, High Risk; MR, Medium Risk; SR, Standard Risk.

Interestingly, none of *CRLF2-high* patients resulted to be positive for the *P2RY8-CRLF2* fusion (16/17 were tested) or the *IGH@-CRLF2* translocation (5/17 were tested) and only 1 of 7 tested patients showed a supernumerary X chromosome (Figure 1A and Table 1).

*JAK2* and *CRLF2* mutations were absent in all analyzed cases, while *IL7Rα* mutations were detected in 5 of 107 tested patients (4.7%), but they were not associated with *CRLF2* over-expression. No statistically significant difference was found in the incidence of recurrent T-ALL genetic aberrations (mutations in *NOTCH1* and *FBXW7* genes and *TAL* deletion) in *CRLF2-low* vs. *CRLF2-high* patients (Figure 1A and Supplementary Table S2).

### Prognostic impact of *CRLF2* over-expression at diagnosis

**Figure 2:**
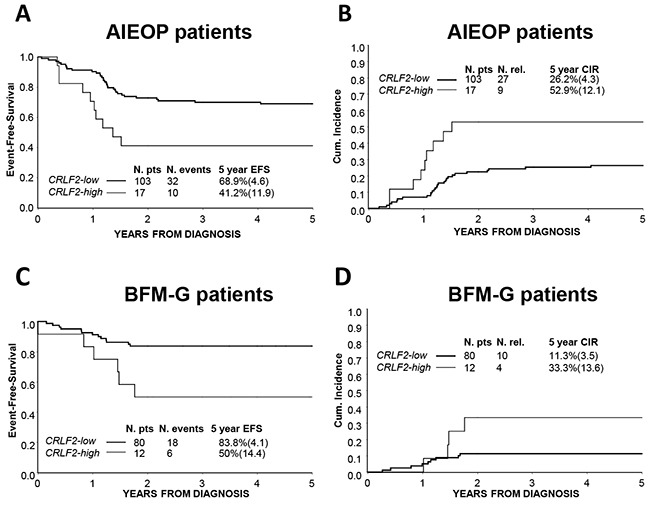
Association of *CRLF2* over-expression to treatment outcome **A.** EFS and **B.** CIR of AIEOP study cohort patients according to *CRLF2* expression: *CRLF2-low* and *CRLF2-high*. **C.** EFS and **D.** CIR of BFM-G study cohort patients according to *CRLF2* expression: *CRLF2-low* and *CRLF2-high*.

*CRLF2-high* AIEOP patients had a significantly lower EFS (41.2%±11.9 vs. 68.9%±4.6, p=0.006) and an increased CIR (52.9%±12.1 vs. 26.2%±4.3, Hazard ratio=2.84, p=0.007) compared to *CRLF2-low* patients (Figure 2A and 2B). Moreover, the 5-year Survival estimates were significantly different, 47.1%±12.1 and 73.8%±4.3, respectively (p=0.009) (Supplementary Figure S2).

In order to validate these results, we analyzed *CRLF2* over-expression in the cohort of 92 consecutive patients treated in German Centers (BFM-G).

Twelve patients (13.0%) were *CRLF2-high* (Figure 1B). Clinical characteristics of BFM-G *CRLF2-high* patients at diagnosis vs. *CRLF2-low* patients are described in Table 1. Unlike *CRLF2-low* patients, a large proportion of *CRLF2-high* patients presented an early-T immunophenotype (6/12 patients, 50.0%; p=<0.001) and in particular 4 out of 6 early-T ALL were classified as ETP-ALL, while no significant differences were observed with respect of sex, age, WBC count, prednisone response, risk group stratification and incidence of recurrent T-ALL genetic aberrations (Table 1, Supplementary Table S2 and Figure 1B). Moreover, similar to what observed in the AIEOP cohort, none of the 92 patients resulted positive for *P2RY8-CRLF2* fusion, while IL7Rα mutations were detected in 8/45 *CRLF2-low* patients and in 2/4 *CRLF2-high* patients (Table 1, Supplementary Table S2 and Figure 1B).

We confirmed in the BFM-G cohort that *CRLF2* over-expression was associated with a significantly worse EFS (50.0%±14.4 vs. 83.8%±4.1, p-value=0.01) and Survival (47.6%±15 vs. 87.5%±3.7, p-value=<0.001) and a higher CIR (33.3%±13.6 vs. 11.3%±3.5, Hazard ratio=3.37, p-value= 0.04) (Figure 2C, 2D and Supplementary Figure S2).

Cox model analysis on 212 patients included in this study (merge AIEOP/BFM-G cohort), was performed to assess the prognostic value of *CRLF2* over-expression after adjusting for final risk stratification. *CRLF2-high* expression had a relevant prognostic impact on the risk of relapse, with a 2.5-fold increase in the risk for positive patients (Hazard ratio 2.47; 95% CI 1.30-4.70; p=0.006), with risk group also maintaining its significant effect (Table 2).

**Table 2:** Cox model on hazard of relapse in AIEOP/BFM-G patient cohort

Characteristics	P-value	Hazard ratio	95% CI
***CRLF2* expression**			
** *CRLF2-low***		1	
** CRLF2-high**	0.006	2.47	1.30-4.70
**Final Risk**			
** No-HR**		1	
** HR**	0.002	2.53	1.41-4.55

**Figure 3:**
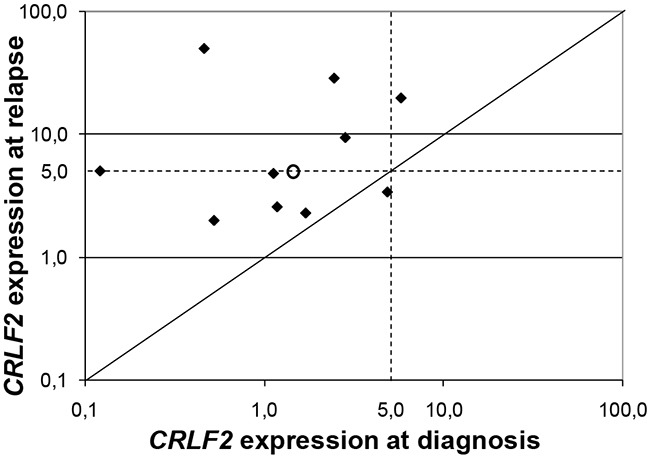
*CRLF2* expression at relapse Log-log plot of the *CRLF2* expression value for 10 paired diagnosis and relapsed specimens. Samples at relapse showed a median value of *CRLF2* expression 3.5 times higher than the respective samples at diagnosis (4.95 vs. 1.43), as indicated with the circle.

Moreover, 10 out of the 34 BM samples collected at relapse from patients in the AIEOP cohort were evaluated for *CRLF2* expression levels. Samples at relapse showed a median value of *CRLF2* expression 3.5 times higher than the respective samples at diagnosis (4.95 vs. 1.43) (Figure 3).

### Outcome and risk group

**Figure 4:**
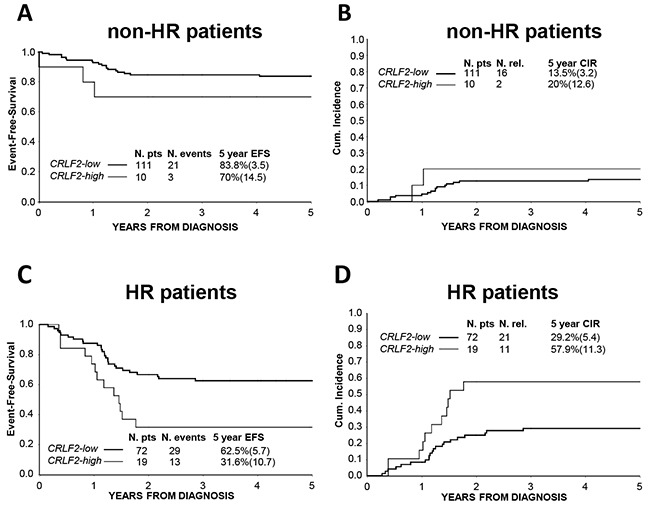
Association of *CRLF2* over-expression to treatment outcome in Risk subgroups **A.** EFS and **B.** CIR of non-HR AIEOP/BFM-G patients according to *CRLF2* expression: *CRLF2-low* and *CRLF2-high*. **C.** EFS and **D.** CIR of HR AIEOP/BFM-G patients according to *CRLF2* expression: *CRLF2-low* and *CRLF2-high*.

We further analyzed the prognostic value of *CRLF2* over-expression jointly in the AIEOP and BFM-G cohorts within non-HR and HR patient subgroups respectively. *CRLF2-high* patients were more frequently allocated to the HR group, being found in 19 out of 91 HR patients (20.9%; p=0.008) vs. 10 out of 121 non-HR patients (8.3%). Only in the HR subgroup, *CRLF2* over-expression was significantly associated with a lower EFS (31.6%±10.7 vs. 62.5%±5.7, p-value=0.01) and a higher CIR (57.9%±11.5 vs. 29.2%±5.4, Hazard ratio =2.70, p-value=0.008) (no-HR: EFS= 70.0%±14.5 vs. 83.8%±3.5, p-value=0.29 and CIR=20.0%±12.6 vs. 13.5%±3.2, Hazard ratio =1.70, p-value=0.48) (Figure 4).

When analyzed according to prednisone response, the majority of *CRLF2-high* patients were PPR (17/29, 59%) (Table 1) and, specifically, 9 of them were allocated to the HR subgroup ‘PPR-only’ (i.e. non-HR by other features: they achieved complete remission after phase IA and did not present high levels of PCR-MRD at day 78). These 9 ‘PPR-only’ among *CRLF2-high* patients were compared with the 36 ‘PPR-only’ within the *CRLF2-low* group; they retained a lower, although not statistically different, EFS (55.6%±16.6 vs. 80.6%±6.6, p-value=0.24), and borderline-significant higher CIR (44.4%±16.6 vs. 11.1%±5.2, Hazard ratio =4.02, p-value=0.05) (Supplementary Figure S3A and S3B).

Moreover, high levels of *CRLF2* were associated with poor outcome also when patients with ETP immunophenotype [26–28] were excluded from the analysis (EFS: 45.8%±10.2 vs. 75.7%±3.2, p-value=<0.001; CIR: 45.8%±10.2 vs. 19.2%±3, Hazard ratio =3.23, p-value=<0.001) (Supplementary Figure S3C and S3D).

In addition, no association between N642H mutation activating STAT5B, abnormality recently identified in T-ALL and associated with a higher risk of relapse, [12] and *CRLF2* over-expression was observed (0/4 STAT5B N642H positive among *CRLF2-high* patients and 1/35 among *CRLF2-low* patients).

### TSLP-induced pSTAT5 response and CRLF2 protein expression

**Figure 5:**
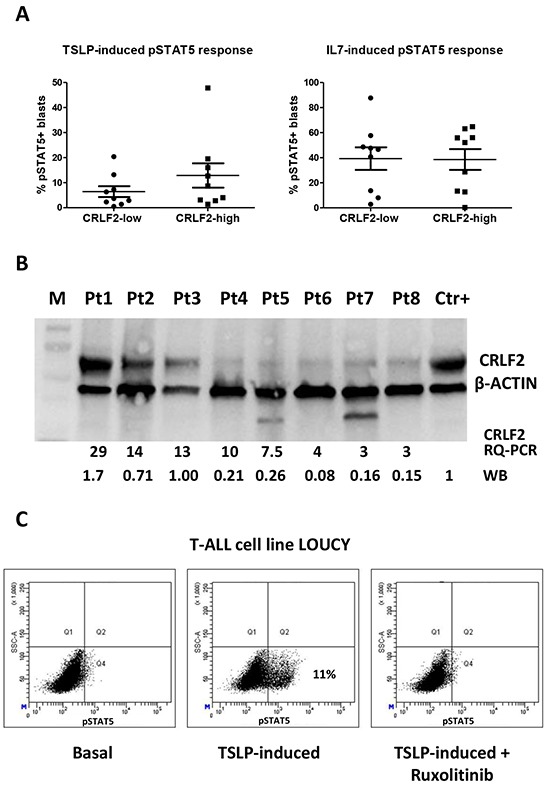
TSLP-induced pSTAT5 response and intracellular expression of CRLF2 **A.** Analysis of TSLP-induced pSTAT5 signaling in 18 T-ALL patients according to their *CRLF2* status: 9 *CRLF2-low* and 9 *CRLF2-high* samples. Distribution of % positive blast cells for pSTAT5 is represented with mean and SEM. Data were normalized to the basal STAT5 phosphorylation status. **B.** Western-blot analysis of CRLF2 and β-ACTIN in T-ALL patients with different *CRLF2* transcript expression levels (indicated in the figure: RQ-PCR). CRLF2 protein expression level was quantified by densitometry, normalized to β-actin, and showed in figure as ratio with respect to the positive control (WB). M: Marker; Ctr+: positive control (BCP-ALL CRLF2+ cell line MHH-CALL4). **C.** Phosphoflow analysis of pSTAT5 in LOUCY cell line. The plots show the % positive cells for pSTAT5 in basal condition and after stimulation with TSLP in absence and in presence of the JAK inhibitor Ruxolitinib.

Eighteen patients (9 *CRLF2-low* and 9 *CRLF2-high*) were subjected to phosphoflow cytometric analysis. We observed a trend (p=0.24) towards a stronger TSLP-induced pSTAT5 response in *CRLF2-high* samples as compared to *CRLF2-low*, showing a mean of 12.89%±4.86 and 6.44%±2.17 of pSTAT5 positive cells, respectively (Figure 5A). This tendency was not observed using IL7 as stimulus (Figure 5A). TSLP-induced pSTAT5 response was specific for blast cells, while it was not observed in normal residual cells (data not shown).

Nine patients, 3 *CRLF2-low* and 6 *CRLF2-high* were also analyzed for CRLF2 surface expression. Unexpectedly, all 9 patients were nearly negative for CRLF2 expression on blast surface (<5% of positive cells, Supplementary Figure S5). By western-blot analysis we confirmed that the protein was translated and observed a correlation between the level of *CRLF2* transcript as measured by RQ-PCR and the protein level (Figure 5B).

In order to experimentally model these results, in collaboration with DSMZ (German Collection of Microorganisms and Cell Cultures GmbH), we tested 24 T-ALL cell lines for the level of *CRLF2* expression. The T-ALL cell line LOUCY presented the highest *CRLF2* expression (Supplementary Figure S5A). As described in the patient cohort, despite the western-blot analysis showed a higher expression of CRLF2 protein in the LOUCY cell line (Supplementary Figure S5B), we observed a very low surface expression of CRLF2 in the *CRLF2-high* LOUCY cells as well as in the other tested cell lines MOLT4, CCRF-CEM, HSB-2 and JURKAT. Interestingly, immunofluorescence analyses confirmed the mainly intracellular localization of CRLF2 in LOUCY cells (Supplementary Figure S6).

Moreover, after TSLP stimulation the *CRLF2-high* LOUCY cells were the only one of the 5 tested cell line showing STAT5 phosphorylation, which was completely inhibited by the JAK inhibitor Ruxolitinib (Figure 5C).

### Gene expression profiling associated with *CRLF2* over-expression

To identify possible transcriptional patterns associated with *CRLF2* over-expression in T-ALL, gene expression analysis was performed. Gene expression profiling (GEP) data were available only for few patients in this study cohort. Therefore, we analyzed T-ALL cases from the same protocol study for whom GEP data were available and representative of the study cohort for clinical features and outcome. Consistent with the 15% *CRLF2-high* cut point, we identified, among 100 GEP arrayed cases, the top 15 with higher *CRLF2* probe values and compared these to the 15 specimens with the lowest expression of *CRLF2*.

As shown in Supplementary Figure S7A, *CRLF2* over-expression was associated with different regulation of 290 genes (link for the list of genes in Supplementary). Notably, gene set enrichment analysis (GSEA) showed an inverse correlation between the expression of *CRLF2* and cell cycle regulators, especially positive regulators (enrichment score= −0.6, P=0.018) (Supplementary Figure S7B).

## DISCUSSION

For the first time, we report here that almost 15% of pediatric T-ALL show overexpression of *CRLF2*, associated to a worse prognosis.

An heterogeneous expression of *CRLF2* was observed among the cohort of 212 T-ALL patients, a distribution comparable to that found in the BCP-ALL cohort [19].

The lowest threshold for *CRLF2* expression showing a significant difference in CIR between two groups was 5 times the median, and this value was then adopted as a cut-point identifying about 15% of patients with *CRLF2* overexpression. Notably, this threshold was much lower that the cut-point adopted for AIEOP BCP-ALL patients (20 times the median value), [19] indicating that T-ALL blast cells might be more sensitive to variation of *CRLF2* expression.

Differently from BCP-ALL, the molecular mechanisms responsible for *CRLF2* over-expression in T-ALL remains to be determined, since none of the tested *CRLF2-high* cases resulted to be positive for *P2RY8-CRLF2* fusion or *IGH@-CRLF2* translocation, and only one showed a supernumerary X chromosome. Indeed, only about 50% of BCP-ALL cases with *high-CRLF2* expression lacked known *CRLF2* genomic lesions [20]. Moreover, while in BCP-ALL *CRLF2* over-expression was frequently associated with mutations in *JAK*, *IL7Rα* and in the same *CRLF2* gene [14, 15, 24, 29, 30], *JAK2* and *CRLF2* mutations were absent in all T-ALL analyzed cases. By contrast, *IL7Rα* mutations were detected in 5/107 T-ALL patients (4.7%). They were all insertions or deletions in the transmembrane domain of the receptor and they were not associated with *CRLF2* over-expression. This last observation is consistent with the results reported in literature, namely that, the IL7Rα mutant protein with insertions did not require CRLF2 for its activation [24].

We show here that *CRLF2* over-expression has a prognostic impact in T-ALL, with *CRLF2-high* patients having a significantly inferior EFS and Survival and a higher CIR compared to *CRLF2-low* patients. The prognostic value of *CRLF2* over-expression, initially identified in the AIEOP cohort, was then confirmed in the BFM-G cohort.

Cox model analysis of the two cohorts analyzed together, adjusted by risk group, showed that *CRLF2-high* expression is an independent prognostic factor in T-ALL, associated with a 2.5-fold increased risk of relapse.

Importantly, as in BCP-ALL [19], also T-ALL samples at relapse showed a median value of *CRLF2* expression higher than the respective samples at diagnosis, this might indicate that blasts with high level of CRLF2, already present at diagnosis in various percentage, are associated with a higher resistance to therapy and are positive selected at relapse or that *CRLF2* expression is gained during treatment.

In order to understand how the prognostic impact of this *CRLF2* alteration can be transferred into clinical practice, *CRLF2* expression was analyzed separately in the different risk subgroups. *CRLF2-high* patients fell more frequently in the HR subgroup (20.9% in HR vs. 8.3% in non-HR), and only in this subgroup, *CRLF2* over-expression was significantly associated with inferior EFS and higher CIR. Therefore, *CRLF2* over-expression identified a subset of HR T-ALL patients with an even dismal outcome.

Among HR cases, most *CRLF2-high* patients were PPR. In detail, among the subgroup of PPR cases lacking other HR features (“PPR-only”), *CRLF2* expression tend to distinguish a different incidence of relapse: 4/9 (44%) in *CRLF2-high* compared to 4/36 (11%) in *CRLF2-low*. Although the low number of patients requires caution in drawing conclusions, if this observation will be confirmed in a large series, *CRLF2-high* could represent a useful marker to identify cases with poor outcome in the still undefined PPR-only subgroup.

The poor outcome of *CRLF2-high* patients is independent of other known prognostic factors, like activating mutations of NOTCH, ETP immunophenotype or STAT5B mutation.

The pathogenetic contribution of *CRLF2* over-expression to T-ALL is still unclear. Interestingly, we observed a tendency to stronger TSLP-induced pSTAT5 response in patients expressing high levels of *CRLF2* transcript, and this finding was confirmed in T-ALL cell lines. Indeed, we observed STAT5 phosphorylation after TSLP stimulation only in LOUCY cells, the T-ALL cell line with the highest level of *CRLF2* transcript expression. Notably, the pSTAT5 response was completely inhibited by the JAK inhibitor Ruxolitinib.

Unexpectedly, although responding to the CRLF2 ligand TSLP, both *CRLF2-low* and *CRLF2-high* T-ALL blats were nearly negative for CRLF2 expression on cell surface. By Western-blot and immunofluorescence analyses, we verified the expression of CRLF2 protein and we observed a correlation between the level of *CRLF2* transcript measured by RQ-PCR and the protein levels. Further biological studies should be afforded to exploit the CRLF2 pathway in T-ALL. Interestingly, it was recently reported in the literature that the activity of another cytokine receptor (cMPL) did not depend on its cell surface expression [31]. The authors assumed that the receptor with an abnormal subcellular distribution may be active and particularly sensitive to the low amount of ligand that may enter into the cell through trace levels of the receptor on the cell surface. It will be important to explore whether this is also the case for CRLF2 to better understand the mechanism of activity of *CRLF2* in T-ALL pathology and to develop strategies for effective leukemia eradication. Our results suggest that, although a direct targeting of CRLF2 on cell surface is not feasible in T-ALL, the downstream JAK/STAT5 signaling could be a potential target for the therapy of this high risk leukemia subgroup.

Finally, by GEP analysis, we found an inverse correlation between expression of *CRLF2* and of positive cell cycle regulators, this suggesting that *CRLF2-high* blasts could have a low proliferating activity and therefore be less sensitive to conventional chemotherapy. Further studies are necessary to test this assumption and to understand whether the unfavorable prognostic role found for *CRLF2* over-expression in T-ALL is due to gene expression alteration and/or to a higher TSLP-induced pSTAT5 response.

In conclusion, we show here that *CRLF2* over-expression is a poor prognostic marker in T-ALL, identifying a subset of HR T-ALL patients that could be eligible for alternative therapies, including those that interfere with the activation of JAK/STAT5 signaling pathway. A potential benefit of hematopoietic stem cell transplantation, and/or innovative drugs for patients with T-ALL with *CRLF2* over-expression needs to be investigated.

## MATERIALS AND METHODS

### Patients

One hundred and twenty T-ALL patients, consecutively enrolled in the AIEOP-BFM ALL 2000 protocol and treated in AIEOP Centers from September 2000 to July 2005, were included in the study as a test cohort. T-ALL diagnosis was performed according to standard cytomorphology, cytochemistry and immunophenotypic criteria. DNA and RNA were isolated from mononuclear cells and cDNA was synthesized according to standard methods [32]. The clinical characteristics of patients analyzed in this study compared to patients enrolled in the same protocol but not analyzed here are shown in Supplementary Table S3. No significant differences were observed with respect to sex, age, white blood cell (WBC) count, immunophenotype, prednisone response, risk group stratification (Supplementary Table S3) and event-free survival (EFS) (Supplementary Figure S8A).

*CRLF2* expression was analyzed in the whole Italian cohort of 120 patients at diagnosis and *P2RY8-CRLF2* rearrangement was tested in 106 patients for which RNA was available. *IGH@-CRLF2* translocation was screened in 5 out of 17 patients positive for *CRLF2* over-expression (≥5 times higher than overall median, see the Results section). DNA was available from 115 patients and the following were analyzed: *CRLF2* mutations (in 84 patients), *IL7Rα* mutations (in 107 patients), *JAK2* mutations (in 90 patients), *SIL-TAL* (DB1) fusion (in 115 patients), *NOTCH1* mutations (in 81 patients) and *FBXW7* mutations (in 91 patients). *CRLF2* expression was also analyzed in 10/34 paired diagnosis and relapse samples for which material was available.

In addition, 92 consecutive patients enrolled in the AIEOP-BFM ALL 2000 study and treated in German Centers (BFM-G) from January 2001 to December 2004 were analyzed as a validation cohort.

The clinical characteristics of the German patients analyzed in this study compared to those not analyzed are shown in Supplementary Table S3 : more patients with a higher WBC count at diagnosis (≥ 100,000/μl: 56.5% vs. 29.7%, p=<0.001) and less with early T-ALL phenotype (15.2% vs. 26.3%, p=0.01) were included in the analysis. However, no significant differences were observed with respect to EFS (Supplementary Figure S8B). *CRLF2* expression was analyzed in the whole BFM-G cohort of 92 patients at diagnosis, and *P2RY8-CRLF2* rearrangement was tested in 90 patients for which RNA was available. *IL7Rα*, *NOTCH1* and *FBXW7* mutations were analyzed in 49 patients from whom DNA was available.

Informed consent to participate in the study was obtained for all patients from parents or legal guardians. Details on risk group definitions and final stratification, treatment outlines, were previously reported [1, 2] and briefly summarized in Supplementary.

### Quantitative expression of CRLF2

*CRLF2* transcript levels on AIEOP and BFM-G samples were centrally analyzed by RQ-PCR using the TaqMan Gene Expression Assay Hs00913509_s1 (Applied Biosystems, Foster City, CA, US), [19] the housekeeping *GUS* gene transcript was tested as an internal control by using the Universal Probe Library (UPL) system (Roche Diagnostics, Basel, Switzerland), following the manufacturers' instructions. Optimal primers and probe for *GUS* amplification were selected using the Roche ProbeFinder software (https://www.roche-appliedscience.com/sis/rtpcr/upl). Each cDNA sample (20ng RNA equivalent) was tested in duplicate (Ct range between replicates <1.5). The amplification reaction was performed on the *7900HT FAST Real Time PCR System* instrument (Applied Biosystems) with the following protocol: initial step at 95°C for 10min, then 50 cycles at 95°C for 15s and at 60°C for 1min

Relative gene expression (indicated as *fold change*) was quantified by the 2^−DDCt^ method [33]. The DDCt for AIEOP and BFM-G samples was referred to the median DCt of their respective cohort.

### CRLF2 expression on cell surface

To assess CRLF2 expression on the surface of T-ALL blasts the following combination of antibodies was used: CRLF2PE (Clone 1B4, Biolegend, London, UK) [24] or isotype matched IgG (Biolegend), CD45PerCP (BD Biosciences, Franklin Lakes, NJ, USA) and CD7ECD (Beckman Coulter, Brea, California, USA). Leukemic blasts were gated as CD45 intermediate/CD7+. The T-ALL cell lines were stained only with the CRLF2PE or the isotype antibody.

### Phosphoflow cytometry assay

Thawed mononuclear cells from primary ALL samples and T-ALL cell lines were starved in X-vivo medium for 2 hours, then cells were stimulated with rhTSLP (100 ng/mL, ImmunoTools, Friesoythe, Germany) or IL-7 (100 ng/mL) for 30 minutes at 37°C to allow signal transduction. To test for sensitivity, the LOUCY cell line, after starvation, was incubated for 24h with Ruxolitinib (Selleck Chemicals, Huston, USA) at 0.5 uM. Cells were fixed and permeabilized and then incubated with surface antigen-directed antibodies and with the anti-phospho-protein-directed antibody p-STAT5 (Y694) AlexaFluor488 (BD Biosciences) or isotype matched IgG (Cell Signaling, Danvers, MA, USA). Cells were examined on a FACSaria™ flow cytometer (BD) and data were collected and analyzed using DIVA™ software (BD). Basal levels of each phosphoprotein was calculated as proportion (%) of phosphoprotein positive (p-positive) cells in basal conditions. Response to each cytokine (rhTSLP or IL-7) was calculated as a difference between the percentage of p-positive cells after exposure to cytokine and the percentage of p-positive cells in the basal state [23].

### Immunoblotting

Western blot analysis of CRLF2 protein was performed by lysing cells in highsalt RIPA buffer (1% NP-40, 0.5% Na-Deoxycholate, 0.1% SDS, 350nM NaCl in PBS) with Protease inhibitor cocktail (Sigma-Aldrich, St. Louis, MO, USA). Goat anti-human CRLF2 antibody (AF981, R&D Systems, Minneapolis, Canada) was used at working dilution 1:2000 and mouse anti-beta-actin antibody at 1:4000 (AC-15, Sigma-Aldrich). Densitometry analyses were performed using Alliance instrument and Uviband software (Uvitec Cambridge, UK).

### Gene-expression and gene set enrichment analysis

All microarray raw data (CEL files) and probe set signals are available at the National Center for Biotechnology Information Gene Expression Omnibus database (GEO, http://www.ncbi.nlm.nih.gov/geo/), series accession number GSE72623.

Details of the protocol in the Supplementary.

### Statistical analysis

EFS and Survival curves were estimated according to the Kaplan-Meier method, and compared using the log-rank test. Cumulative incidence of relapse/resistance (CIR) was estimated by adjusting for competing risks of other events. The Cox regression model was applied to evaluate the prognostic value of *CRLF2* expression on the cause-specific hazard of relapse/resistance, after adjusting for risk group. Follow-up was updated in January 2014. Analyses were carried out using SAS version 9.2. The study protocol was registered at http://clinicaltrials.gov (NCT00613457 for AIEOP, NCT00430118 for BFM).

## SUPPLEMENTARY METHODS FIGURES AND TABLES



## References

[1] Schrappe M, Valsecchi MG, Bartram CR, Schrauder A, Panzer-Grumayer R, Moricke A, Parasole R, Zimmermann M, Dworzak M, Buldini B, Reiter A, Basso G, Klingebiel T (2011). Late MRD response determines relapse risk overall and in subsets of childhood T-cell ALL: results of the AIEOP-BFM-ALL 2000 study. Blood.

[2] Conter V, Bartram CR, Valsecchi MG, Schrauder A, Panzer-Grumayer R, Moricke A, Arico M, Zimmermann M, Mann G, De Rossi G, Stanulla M, Locatelli F, Basso G (2010). Molecular response to treatment redefines all prognostic factors in children and adolescents with B-cell precursor acute lymphoblastic leukemia: results in 3184 patients of the AIEOP-BFM ALL 2000 study. Blood.

[3] Eckert C, Hagedorn N, Sramkova L, Mann G, Panzer-Grumayer R, Peters C, Bourquin JP, Klingebiel T, Borkhardt A, Cario G, Alten J, Escherich G, Astrahantseff K (2015). Monitoring minimal residual disease in children with high-risk relapses of acute lymphoblastic leukemia: prognostic relevance of early and late assessment. Leukemia.

[4] Izraeli S (2010). Application of genomics for risk stratification of childhood acute lymphoblastic leukaemia: from bench to bedside?. Br J Haematol.

[5] Schultz KR, Bowman WP, Aledo A, Slayton WB, Sather H, Devidas M, Wang C, Davies SM, Gaynon PS, Trigg M, Rutledge R, Burden L, Jorstad D (2009). Improved early event-free survival with imatinib in Philadelphia chromosome-positive acute lymphoblastic leukemia: a children's oncology group study. J Clin Oncol.

[6] Ferrando AA, Neuberg DS, Staunton J, Loh ML, Huard C, Raimondi SC, Behm FG, Pui CH, Downing JR, Gilliland DG, Lander ES, Golub TR, Look AT (2002). Gene expression signatures define novel oncogenic pathways in T cell acute lymphoblastic leukemia. Cancer Cell.

[7] Kox C, Zimmermann M, Stanulla M, Leible S, Schrappe M, Ludwig WD, Koehler R, Tolle G, Bandapalli OR, Breit S, Muckenthaler MU, Kulozik AE (2010). The favorable effect of activating NOTCH1 receptor mutations on long-term outcome in T-ALL patients treated on the ALL-BFM 2000 protocol can be separated from FBXW7 loss of function. Leukemia.

[8] La Starza R, Lettieri A, Pierini V, Nofrini V, Gorello P, Songia S, Crescenzi B, Te Kronnie G, Giordan M, Leszl A, Valsecchi MG, Aversa F, Basso G (2013). Linking genomic lesions with minimal residual disease improves prognostic stratification in children with T-cell acute lymphoblastic leukaemia. Leuk Res.

[9] Milani G, Rebora P, Accordi B, Galla L, Bresolin S, Cazzaniga G, Buldini B, Mura R, Ladogana S, Giraldi E, Conter V, Te Kronnie G, Valsecchi MG (2014). Low PKCalpha expression within the MRD-HR stratum defines a new subgroup of childhood T-ALL with very poor outcome. Oncotarget.

[10] Breit S, Stanulla M, Flohr T, Schrappe M, Ludwig WD, Tolle G, Happich M, Muckenthaler MU, Kulozik AE (2006). Activating NOTCH1 mutations predict favorable early treatment response and long-term outcome in childhood precursor T-cell lymphoblastic leukemia. Blood.

[11] Bandapalli OR, Zimmermann M, Kox C, Stanulla M, Schrappe M, Ludwig WD, Koehler R, Muckenthaler MU, Kulozik AE (2013). NOTCH1 activation clinically antagonizes the unfavorable effect of PTEN inactivation in BFM-treated children with precursor T-cell acute lymphoblastic leukemia. Haematologica.

[12] Bandapalli OR, Schuessele S, Kunz JB, Rausch T, Stutz AM, Tal N, Geron I, Gershman N, Izraeli S, Eilers J, Vaezipour N, Kirschner-Schwabe R, Hof J (2014). The activating STAT5B N642H mutation is a common abnormality in pediatric T-cell acute lymphoblastic leukemia and confers a higher risk of relapse. Haematologica.

[13] Fogelstrand L, Staffas A, Wasslavik C, Sjogren H, Soderhall S, Frost BM, Forestier E, Degerman S, Behrendtz M, Heldrup J, Karrman K, Johansson B, Heyman M (2014). Prognostic implications of mutations in NOTCH1 and FBXW7 in childhood T-ALL treated according to the NOPHO ALL-1992 and ALL-2000 protocols. Pediatr Blood Cancer.

[14] Russell LJ, Capasso M, Vater I, Akasaka T, Bernard OA, Calasanz MJ, Chandrasekaran T, Chapiro E, Gesk S, Griffiths M, Guttery DS, Haferlach C, Harder L (2009). Deregulated expression of cytokine receptor gene CRLF2 is involved in lymphoid transformation in B-cell precursor acute lymphoblastic leukemia. Blood.

[15] Mullighan CG, Collins-Underwood JR, Phillips LA, Loudin MG, Liu W, Zhang J, Ma J, Coustan-Smith E, Harvey RC, Willman CL, Mikhail FM, Meyer J, Carroll AJ (2009). Rearrangement of CRLF2 in B-progenitor- and Down syndrome-associated acute lymphoblastic leukemia. Nat Genet.

[16] Cario G, Zimmermann M, Romey R, Gesk S, Vater I, Harbott J, Schrauder A, Moericke A, Izraeli S, Akasaka T, Dyer MJ, Siebert R, Schrappe M (2010). Presence of the P2RY8-CRLF2 rearrangement is associated with a poor prognosis in non-high-risk precursor B-cell acute lymphoblastic leukemia in children treated according to the ALL-BFM 2000 protocol. Blood.

[17] Ensor HM, Schwab C, Russell LJ, Richards SM, Morrison H, Masic D, Jones L, Kinsey SE, Vora AJ, Mitchell CD, Harrison CJ, Moorman AV (2011). Demographic clinical and outcome features of children with acute lymphoblastic leukemia and CRLF2 deregulation: results from the MRC ALL97 clinical trial. Blood.

[18] Harvey RC, Mullighan CG, Chen IM, Wharton W, Mikhail FM, Carroll AJ, Kang H, Liu W, Dobbin KK, Smith MA, Carroll WL, Devidas M, Bowman WP (2010). Rearrangement of CRLF2 is associated with mutation of JAK kinases, alteration of IKZF1 Hispanic/Latino ethnicity and a poor outcome in pediatric B-progenitor acute lymphoblastic leukemia. Blood.

[19] Palmi C, Vendramini E, Silvestri D, Longinotti G, Frison D, Cario G, Shochat C, Stanulla M, Rossi V, Di Meglio AM, Villa T, Giarin E, Fazio G (2012). Poor prognosis for P2RY8-CRLF2 fusion but not for CRLF2 over-expression in children with intermediate risk B-cell precursor acute lymphoblastic leukemia. Leukemia.

[20] Chen IM, Harvey RC, Mullighan CG, Gastier-Foster J, Wharton W, Kang H, Borowitz MJ, Camitta BM, Carroll AJ, Devidas M, Pullen DJ, Payne-Turner D, Tasian SK (2012). Outcome modeling with CRLF2 IKZF1 JAK and minimal residual disease in pediatric acute lymphoblastic leukemia: a Children's Oncology Group study. Blood.

[21] Liu YJ, Soumelis V, Watanabe N, Ito T, Wang YH, Malefyt Rde W, Omori M, Zhou B, Ziegler SF (2007). TSLP: an epithelial cell cytokine that regulates T cell differentiation by conditioning dendritic cell maturation. Annual review of immunology.

[22] Rochman Y, Kashyap M, Robinson GW, Sakamoto K, Gomez-Rodriguez J, Wagner KU, Leonard WJ (2010). Thymic stromal lymphopoietin-mediated STAT5 phosphorylation via kinases JAK1 and JAK2 reveals a key difference from IL-7-induced signaling. Proc Natl Acad Sci U S A.

[23] Bugarin C, Sarno J, Palmi C, Savino AM, Te Kronnie G, Dworzak M, Schumich A, Buldini B, Maglia O, Sala S, Bronzini I, Bourquin JP, Mejstrikova E (2015). Fine Tuning of Surface CRLF2 Expression and Its Associated Signaling Profile in Childhood B Cell Precursor Acute Lymphoblastic Leukemia. Haematologica.

[24] Shochat C, Tal N, Bandapalli OR, Palmi C, Ganmore I, te Kronnie G, Cario G, Cazzaniga G, Kulozik AE, Stanulla M, Schrappe M, Biondi A, Basso G (2011). Gain-of-function mutations in interleukin-7 receptor-alpha (IL7R) in childhood acute lymphoblastic leukemias. J Exp Med.

[25] Zenatti PP, Ribeiro D, Li W, Zuurbier L, Silva MC, Paganin M, Tritapoe J, Hixon JA, Silveira AB, Cardoso BA, Sarmento LM, Correia N, Toribio ML (2011). Oncogenic IL7R gain-of-function mutations in childhood T-cell acute lymphoblastic leukemia. Nat Genet.

[26] Coustan-Smith E, Mullighan CG, Onciu M, Behm FG, Raimondi SC, Pei D, Cheng C, Su X, Rubnitz JE, Basso G, Biondi A, Pui CH, Downing JR (2009). Early T-cell precursor leukaemia: a subtype of very high-risk acute lymphoblastic leukaemia. Lancet Oncol.

[27] Patrick K, Wade R, Goulden N, Mitchell C, Moorman AV, Rowntree C, Jenkinson S, Hough R, Vora A (2014). Outcome for children and young people with Early T-cell precursor acute lymphoblastic leukaemia treated on a contemporary protocol UKALL 2003. Br J Haematol.

[28] Conter V, Valsecchi MG, Buldini B, Parasole R, Locatelli F, Colombini A, Rizzari C, Putti MC, Barisone E, Nigro LL, Santoro N, Ziino O, Pession A (2016). Early T-cell precursor acute lymphoblastic leukaemia in children treated in AIEOP centres with AIEOP-BFM protocols: a retrospective analysis. The Lancet Haematology.

[29] Hertzberg L, Vendramini E, Ganmore I, Cazzaniga G, Schmitz M, Chalker J, Shiloh R, Iacobucci I, Shochat C, Zeligson S, Cario G, Stanulla M, Strehl S (2010). Down syndrome acute lymphoblastic leukemia a highly heterogeneous disease in which aberrant expression of CRLF2 is associated with mutated JAK2: a report from the International BFM Study Group. Blood.

[30] Yoda A, Yoda Y, Chiaretti S, Bar-Natan M, Mani K, Rodig SJ, West N, Xiao Y, Brown JR, Mitsiades C, Sattler M, Kutok JL, DeAngelo DJ (2010). Functional screening identifies CRLF2 in precursor B-cell acute lymphoblastic leukemia. Proc Natl Acad Sci U S A.

[31] Stockklausner C, Klotter AC, Dickemann N, Kuhlee IN, Duffert CM, Kerber C, Gehring NH, Kulozik AE (2015). The thrombopoietin receptor P106L mutation functionally separates receptor signaling activity from thrombopoietin homeostasis. Blood.

[32] van Dongen JJ, Macintyre EA, Gabert JA, Delabesse E, Rossi V, Saglio G, Gottardi E, Rambaldi A, Dotti G, Griesinger F, Parreira A, Gameiro P, Diaz MG (1999). Standardized RT-PCR analysis of fusion gene transcripts from chromosome aberrations in acute leukemia for detection of minimal residual disease Report of the BIOMED-1 Concerted Action: investigation of minimal residual disease in acute leukemia. Leukemia.

[33] Livak KJ, Schmittgen TD (2001). Analysis of relative gene expression data using real-time quantitative PCR and the 2(-Delta Delta C(T)) Method. Methods.

