# Supplementary material for: CRLF2 over-expression is a poor prognostic marker in children with high risk T-cell acute lymphoblastic leukemia
Source: Oncotarget. 2016 Jul 15;7(37):59260–72. doi: 10.18632/oncotarget.10610 (PMC5312310; doi:10.18632/oncotarget.10610)
Supplement: Supplementary file 1 [file oncotarget-07-59260-s001.pdf]

CRLF2 Over-expression is a poor prognostic marker in children with high risk t-cell acute lymphoblastic leukemia

SUPPLEMENTARY METHODS

Protocol stratification

Patient risk groups were defined as follows. The High Risk (HR) group included patients with prednisone poor response (≥ 1,000 blasts/μL on day 8 peripheral blood after 7 days of prednisone and one dose of intrathecal methotrexate on day 1) orinability to achieve clinical remission after Induction Phase IA; high burden (≥ 10^{-3}) of PCR-Minimal Residual Disease (MRD) at day 78. The non-HR group consisting of Standard Risk (SR) and Medium Risk (MR). The SR group included patients who lacked high-risk criteria and tested negative to PCR-MRD for two sensitive markers (≥ 1& $\times 10^{-4}$) at both day 33 and day 78. The MR group included the remaining patients, and those not evaluated by PCR-MRD.

PCR-MRD was detected by RQ-PCR of Immunoglobulin and/or T-cell receptor gene rearrangements in bone marrow samples collected at the end of the IA (TP1, day 33), and IB (TP2, day 78) induction phases [1]; data were interpreted according to EuroMRD guidelines [2].

In the protocol AIEOP-BFM ALL 2000 stratification criteria were common for AIEOP and BFM-G, but, as reported, there were some differences in treatment (slightly less intensive in AIEOP). [1] These could interfere with the outcome that indeed is inferior in AIEOP T-ALL patients to that observed in BFM-G (10 years EFS 65.6% versus 73.4%; 10 years survival 72% versus 79%) (manuscript in preparation).

CRLF2 rearrangements

The presence of the *P2RY8-CRLF2* fusion transcript in the AIEOP and BFM-G cohort was investigated by RQ-PCR, as previously described. [3] In particular, the UPL System was used, with primers designed in the first exon of *P2RY8* (5'-GCTACTTCTGCCGCTGCTT-3') and in the first exon of *CRLF2* (5'-GCAGAAAGACGGCAGCTC-3') with the UPL probe n. 28 (Roche UPL cat. n. 04687604001).

IGH@-CRLF2 translocationor any other rearrangement involving *CRLF2* was searched in *CRLF2* over-expressed AIEOP patient, for which fixed cells from BM at diagnosis were available, by Fluorescence in situ hybridization (FISH) on interphase nuclei using CRLF2 Breakapart Probe (Cytocell Ltd, Cambridge, UK). Analyses were carried out using Zeiss Axio Imager Z2 fluorescent microscope (Carl Zeiss AG Corporate, Oberkochen, Germany) and ISIS software (MetaSystems GmbH, Altlussheim, Germany). For each case 150/200 interphase nuclei were scored.

Other genetic aberrations

High Resolution Melting (HRM) analysis was performed to identify *JAK2* mutations in exon 16, as previously described [4].

Sequencing of *NOTCH1*, *FBXW7* and *CRLF2* was performed by PCR amplification and direct sequencing. Whole genome was DNA amplified using the GenomePhi V2 DNA Amplification Kit (GE Heathcare Life Science). The following primers were designed for *CRLF2*: *CRLF2-F* (5'-GTGGGCATTGTATGGAAACTGA-3') and *CRLF2-R* (5'-GAGACTGGTTAGGGATGAGAT GT-3'); while previously reported primers were used for *NOTCH1* and *FBXW7* [5] [6].

Cell culture

The human T-ALL cell lines LOUCY (a kind gift of DSMZ, Germany), MOLT-4, CCRF-CEM and Jurkat were cultured in RPMI medium with 10-20% bovine calf serum.

Immunofluorescence-analyses

Cells were fixed in 4% paraformaldehyde, resuspended in PBS, and let to dry out at RT on gelatin-coated slides. After re-hydration, samples were treated briefly with 0,1 M Glycin in PBS (pH 7.4) followed by 0.3% Triton X-100 buffer. Cells were incubated O/N at 4°C with primary Abs, (goat anti-human CRLF2 antibody AF981, R&D Systems and rabbit anti-Calnexin, Stressgen, Victoria, Canada) washed and incubated for 1h at RT with secondary Abs (488-Donkey anti-goat, Abcam, Cambridge, UK and 555-Donkey anti-rabbit, Immunological Sciences, Rome, Italy) and 594-WGA (Life Technologies, Carlsbad, CA, USA). For nuclei staining slides were treated with Toto-3 iodide 642/660 (Life Technologies). Confocal microscopy was carried out on a Radiance 2100 microscope (Biorad Laboratories, Hercules, CA, USA) equipped with a Red laser diode and Kripon/Argon laser.

Gene-expression and gene set enrichment analysis

An independent cohort was used to perform Gene expression profiling (GEP) analysis. RNA samples of 100 T-ALL (AIEOP ALL study cohort, diagnosed from 2000 to 2006) were processed according to Affymetrix protocols as previously described.[7] GeneChip Human Genome U133 Plus 2.0 array were used and microarray data (.CEL files) were generated from raw signals using integrated microarray Affymetrix software. Microarray data, normalized by the justRMA algorithm, were analyzed by R-Bioconductor (Version 2.15.3). The expression values of *CRLF2* probe 208303_s_t were analyzed in the 100 T-ALL specimens using the same cut-off values as previously established for *CRLF2* RQ-PCR expression values. Using all gene expression data of .CEL files the 15% of patients with highest *CRLF2* expression were compared with the 15% of patients with lowest *CRLF2* expression. Differentially expressed probes between the two groups (*CRLF2-high* vs. *CRLF2-low*) groups were obtained using Wilcoxon T-test and local false discovery rate (lfdr) was used to correct the p-value. A lfdr <0.05 was considered significant for probe sets differentially expressed between compared groups.

Gene set enrichment analysis (GSEA) was run on the differentially expressed genes resulting from the Wilcoxon test in order to explore presence of specific pathways and oncogenic signature defined directly from microarray gene expression data.

REFERENCES

1. Schrappe M, Valsecchi MG, Bartram CR, Schrauder A, Panzer-Grumayer R, Moricke A, Parasole R, Zimmermann M, Dworzak M, Buldini B, Reiter A, Basso G, Klingebiel T, Messina C, Ratei R, Cazzaniga G, et al. Late MRD response determines relapse risk overall and in subsets of childhood T-cell ALL: results of the AIEOP-BFM-ALL 2000 study. Blood. 2011; 118:2077-2084.
2. van der Velden VH, Cazzaniga G, Schrauder A, Hancock J, Bader P, Panzer-Grumayer ER, Flohr T, Sutton R, Cave H, Madsen HO, Cayuela JM, Trka J, Eckert C, Foroni L, Zur Stadt U, Beldjord K, et al. Analysis of minimal residual disease by Ig/TCR gene rearrangements: guidelines for interpretation of real-time quantitative PCR data. Leukemia. 2007; 21:604-611.
3. Bugarin C, Sarno J, Palmi C, Savino AM, Te Kronnie G, Dworzak M, Schumich A, Buldini B, Maglia O, Sala S, Bronzini I, Bourquin JP, Mejstrikova E, Hrusak O, Luria D, Basso G, et al. Fine Tuning of Surface CRLF2 Expression and Its Associated Signaling Profile in Childhood B Cell Precursor Acute Lymphoblastic Leukemia. Haematologica. 2015; 100:e229-232.
4. Palmi C, Vendramini E, Silvestri D, Longinotti G, Frison D, Cario G, Shochat C, Stanulla M, Rossi V, Di Meglio AM, Villa T, Giarin E, Fazio G, Leszl A, Schrappe M, Basso G, et al. Poor prognosis for P2RY8-CRLF2 fusion but not for CRLF2 over-expression in children with intermediate risk B-cell precursor acute lymphoblastic leukemia. Leukemia. 2012; 26:2245-2253.
5. Sulis ML, Williams O, Palomero T, Tosello V, Pallikuppam S, Real PJ, Barnes K, Zuurbier L, Meijerink JP and Ferrando AA. NOTCH1 extracellular juxtamembrane expansion mutations in T-ALL. Blood. 2008; 112:733-740.
6. Thompson BJ, Buonamici S, Sulis ML, Palomero T, Vilimas T, Basso G, Ferrando A and Aifantis I. The SCFFBW7 ubiquitin ligase complex as a tumor suppressor in T cell leukemia. J Exp Med. 2007; 204:1825-1835.
7. Haferlach T, Kohlmann A, Wieczorek L, Basso G, Kronnie GT, Bene MC, De Vos J, Hernandez JM, Hofmann WK, Mills KI, Gilkes A, Chiaretti S, Shurtleff SA, Kipps TJ, Rassenti LZ, Yeoh AE, et al. Clinical utility of microarray-based gene expression profiling in the diagnosis and subclassification of leukemia: report from the International Microarray Innovations in Leukemia Study Group. J Clin Oncol. 2010; 28:2529-2537.

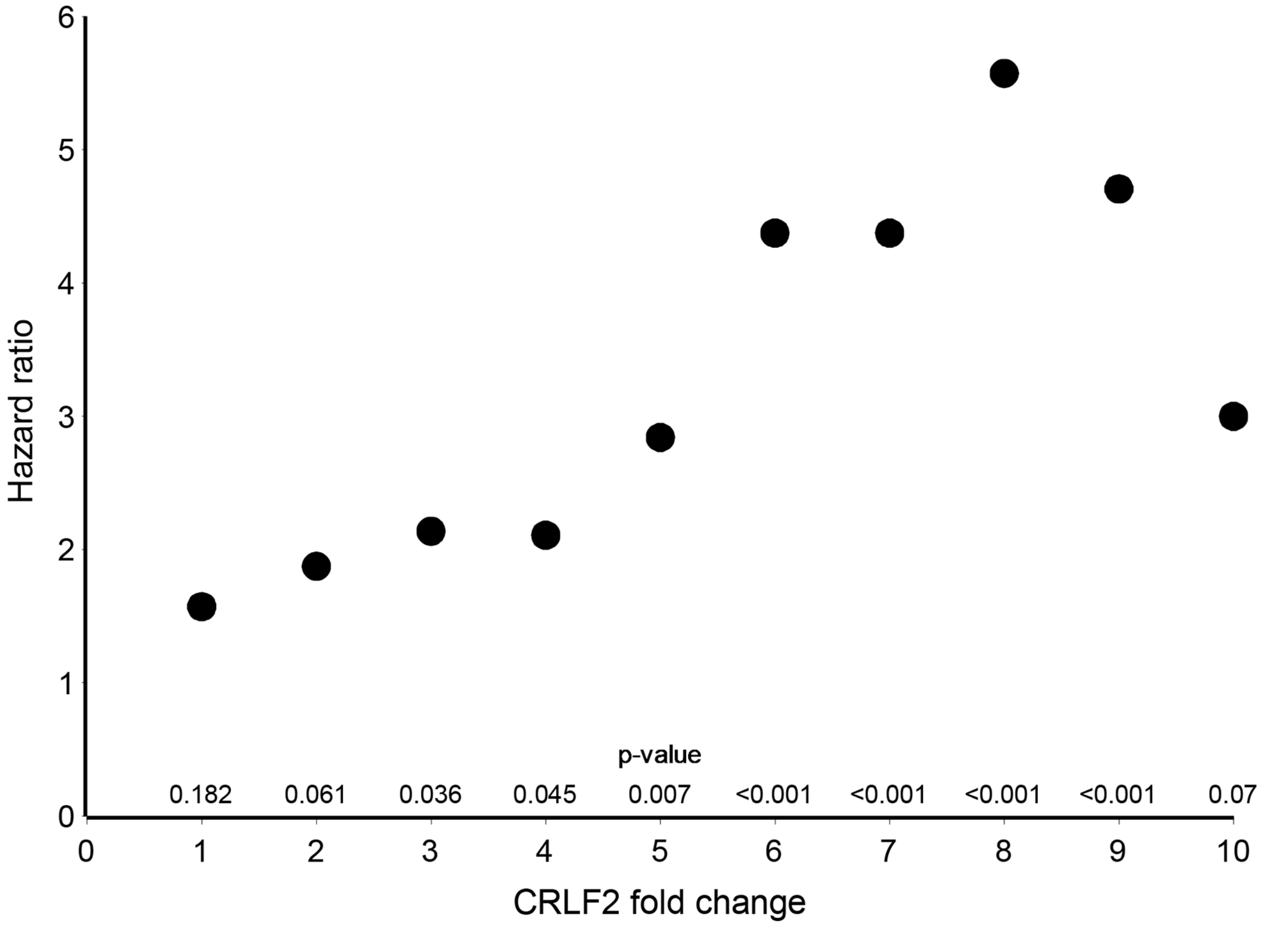

Supplementary Figure S1: CIR hazard ratio associated with different cut-points of *CRLF2* expression. Potential cut-points of *CRLF2* expression were evaluated. The estimated hazard ratios quantified theability to discriminate prognosis in terms of the CIR hazard at each of the cut-points.P-value for each cut-point wereindicated.

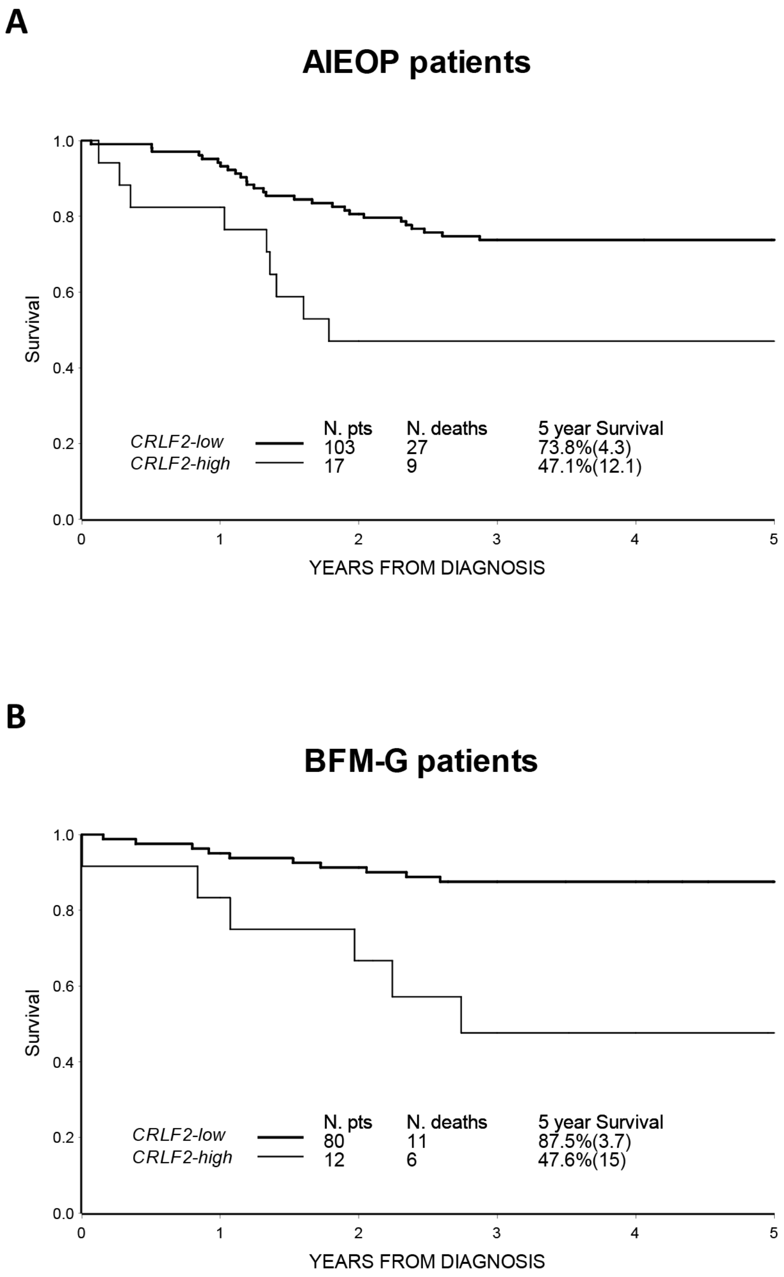

Supplementary Figure S2: Association of *CRLF2* over-expression to overall survival. Overall survival of AIEOP **A.** and BFM-G **B.** study cohort patients according to *CRLF2* expression: *CRLF2-low* and *CRLF2-high*.

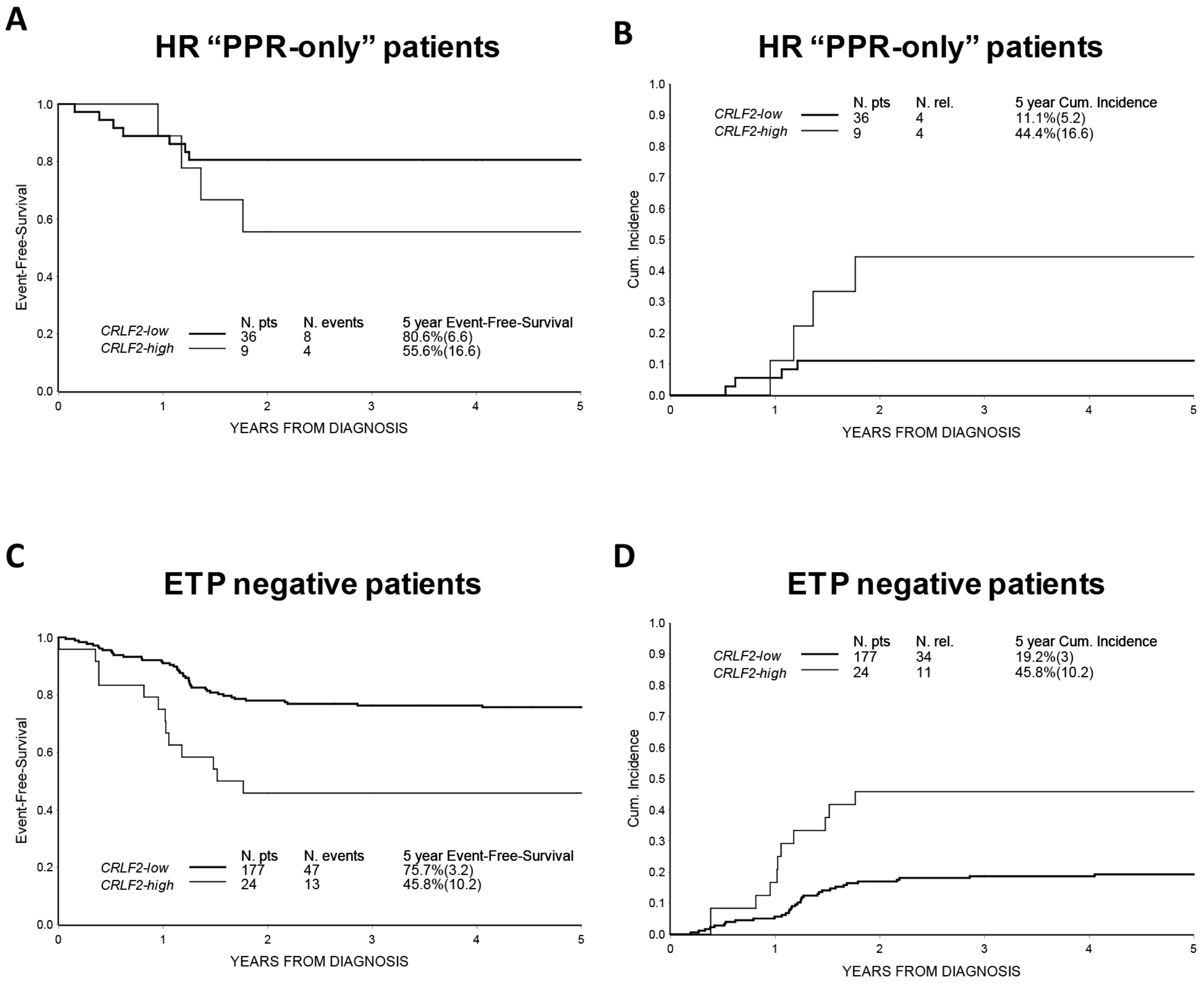

Supplementary Figure S3: Association of *CRLF2* over-expression to treatment outcome in the HR subgroup “PPR-only” or excluding patients with ETP immunophenotype. A. EFS and **B.** CIR of HR “PPR-only” AIEOP/BFM-G patients according to *CRLF2* expression: *CRLF2-low* and *CRLF2-high*. **C.** EFS and **D.** CIR of AIEOP/BFM-G patients according to *CRLF2* expression: *CRLF2-low* and *CRLF2-high* when patients with ETP immunophenotype were excluded from the analysis.

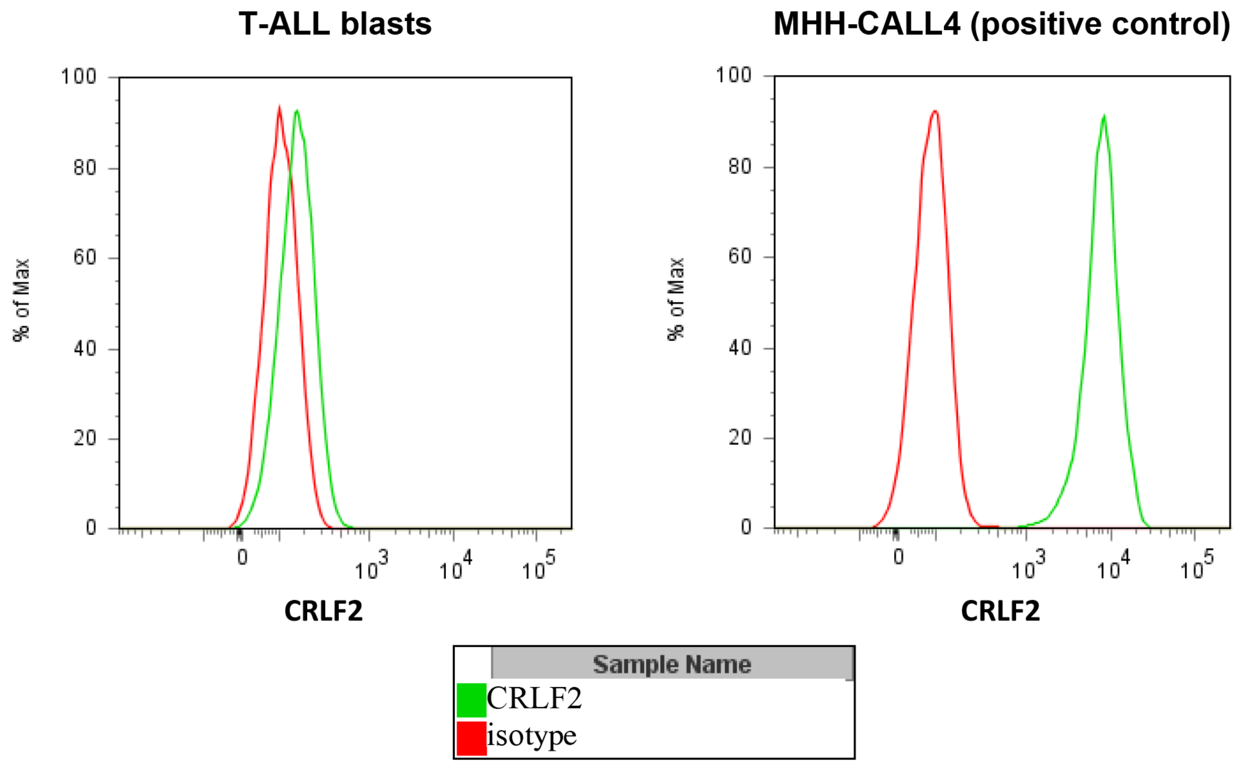

Supplementary Figure S4: CRLF2 expression on cell-surface. Analysis by FACS of CRLF2 expression on cell-surface of T-ALL blasts and of the BCP-ALL cell line MHH-CALL4 (positive control). The figure shows a representative experiment.

A

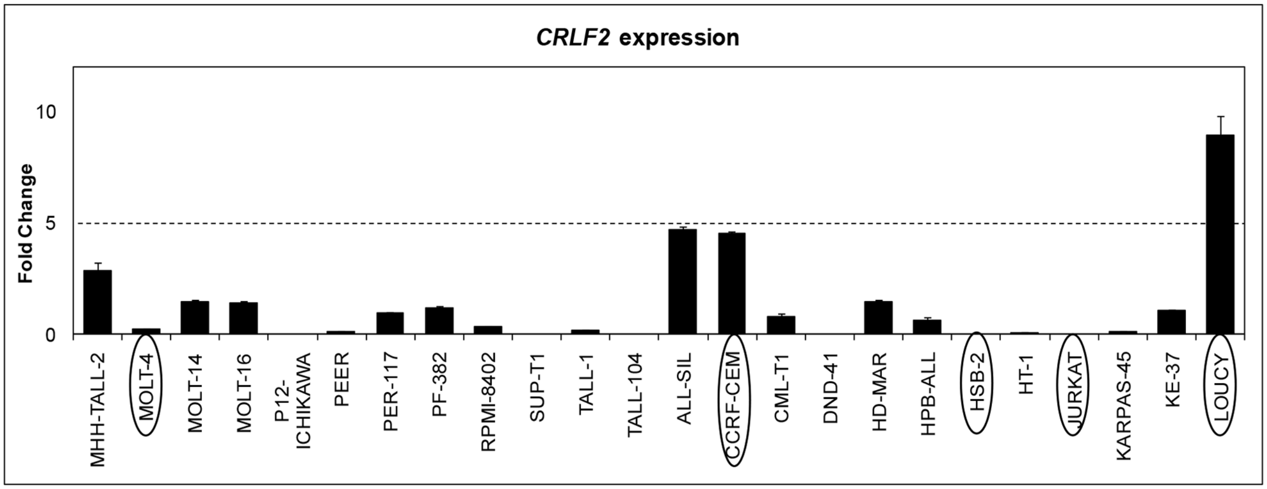

B

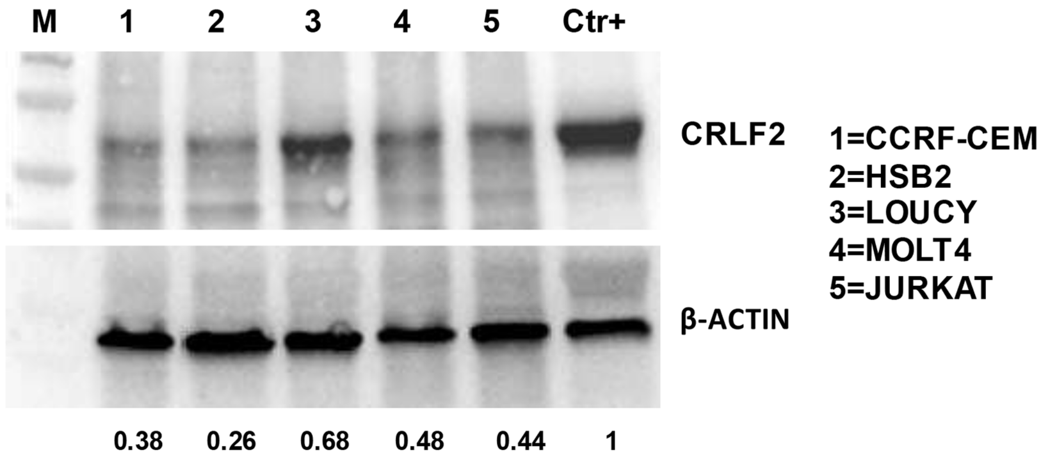

Supplementary Figure S5: CRLF2 transcript and protein expression on T-ALL cell lines. A. RQ-PCR analysis of *CRLF2* expression in 24 T-ALL cell lines. Results are reported as fold changes on the median expression value of all the 24 tested cell lines. The 5 cell lines analyzed furthermore for CRLF2 expression on surface and intra-cellular and for pSTAT5 are circled. **B.** Western-blot analysis of CRLF2 and β-ACTIN in T-ALL cell lines. M: Marker; Ctr+: positive control (BCP-ALL CRLF2+ cell line MHH-CALL4).

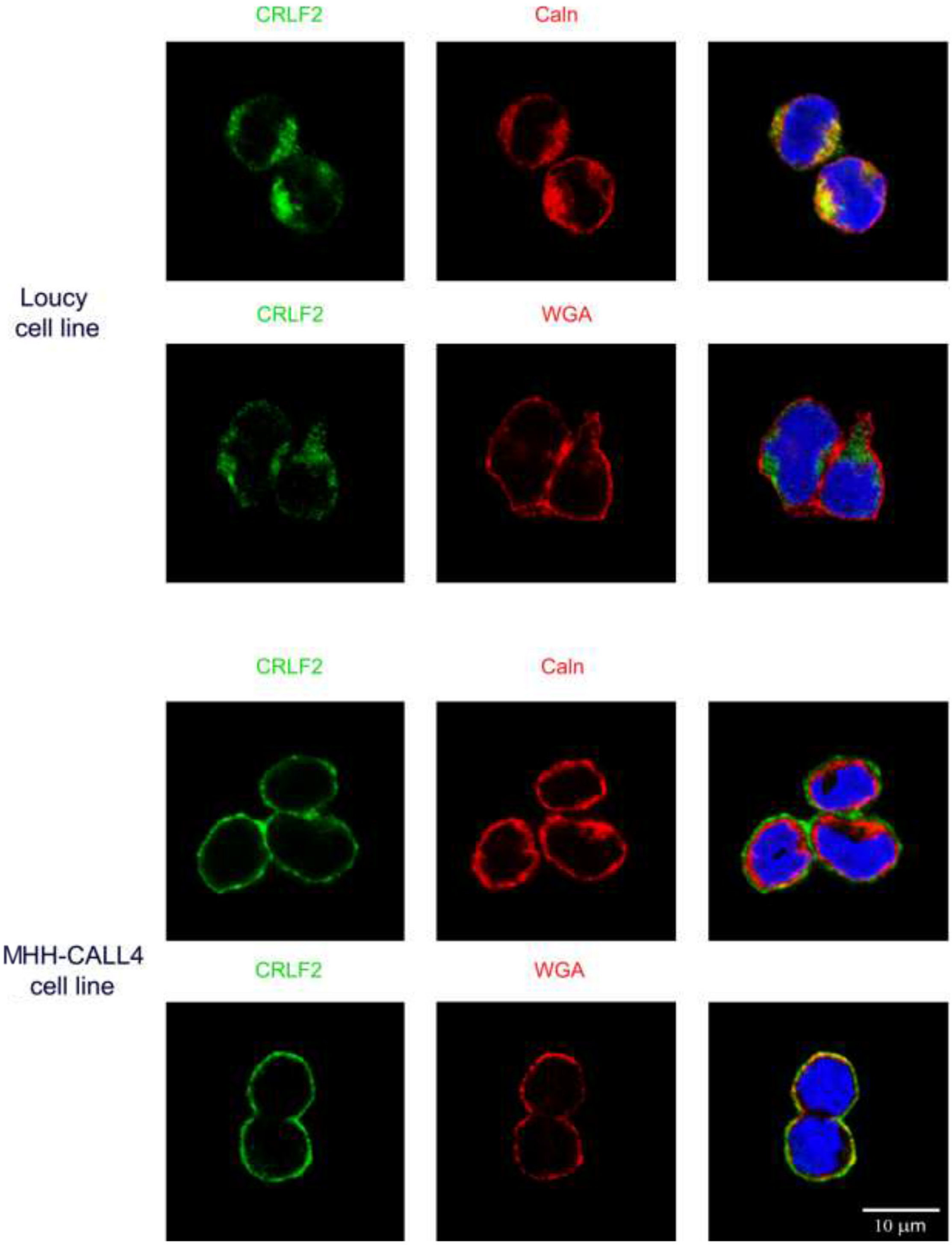

Supplementary Figure S6: Immunofluorescence analysis of LOUCY cell line and MHH-CALL4 cell line (positive control) for CRLF2 localization. Loucy cell line and the BCP-ALL cell line MHH-CALL4 (positive control) were observed by confocal microscope after staining with CRLF2 (green), the endoplasmic reticulum-marker calnexin (Caln) or the surface membrane stain WGA (red) and Toto-3 for the nuclei (blue). The panels on the right represent overlays.

A

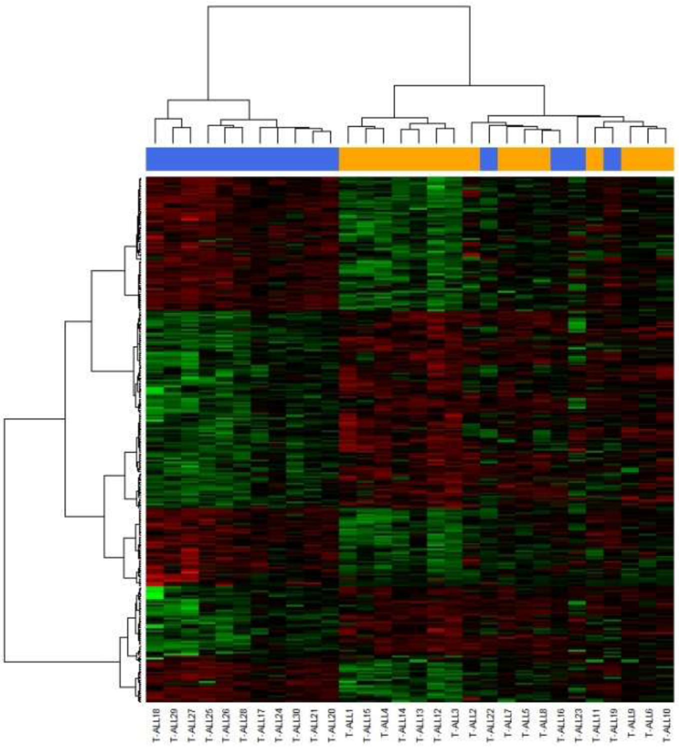

B

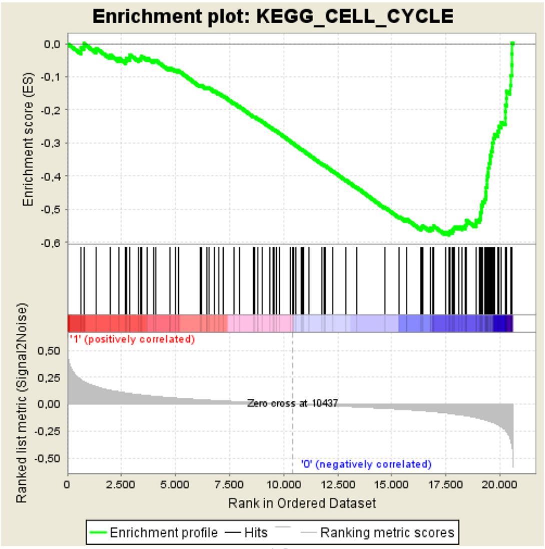

Supplementary Figure S7: Gene expression profiling associated with *CRLF2* over-expression. Since GEP data were not available from patients of this study cohort, T-ALL cases treated according to the same protocol with available GEP data were analyzed. **A.** Consistent with the 15% *CRLF2-high*, identified in the patient cohort, among 100 T-ALL arrayed cases, the top 15 specimens with higher *CRLF2* probe values (orange) were compared to the 15 lowest expressing *CRLF2* specimens (blue). The heat map shows the unsupervised clustering of 290 differentially regulated genes. Red color depicts over-expressed genes, while down-regulated genes are labeled green. **B.** Gene set enrichment analysis (GSEA) showing an inverse correlation between the expression of *CRLF2* and cell cycle regulators (enrichment score= -0.6, P=0.018). Enrichment plots depict enrichment scores (green lines) reflecting the appearance of members of the annotated gene sets (black vertical lines) along the gene list ranked from *CRLF2-low* (red) to *CRLF2-high* (blue).

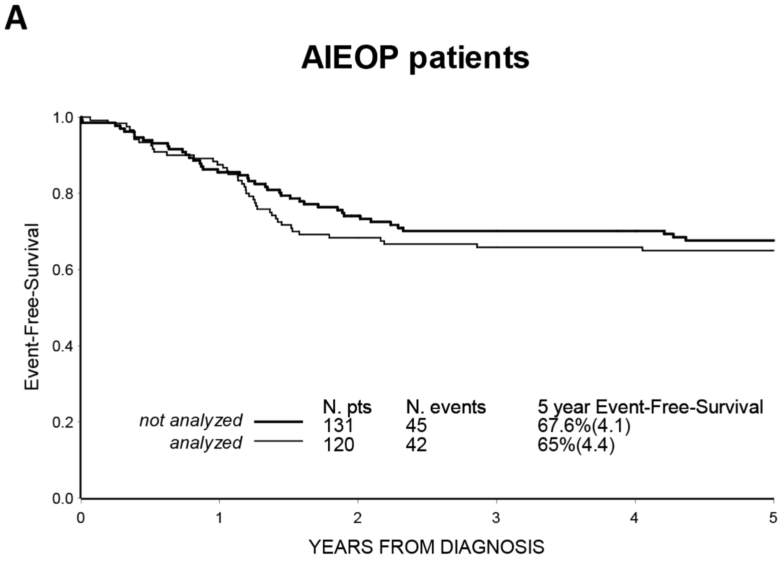

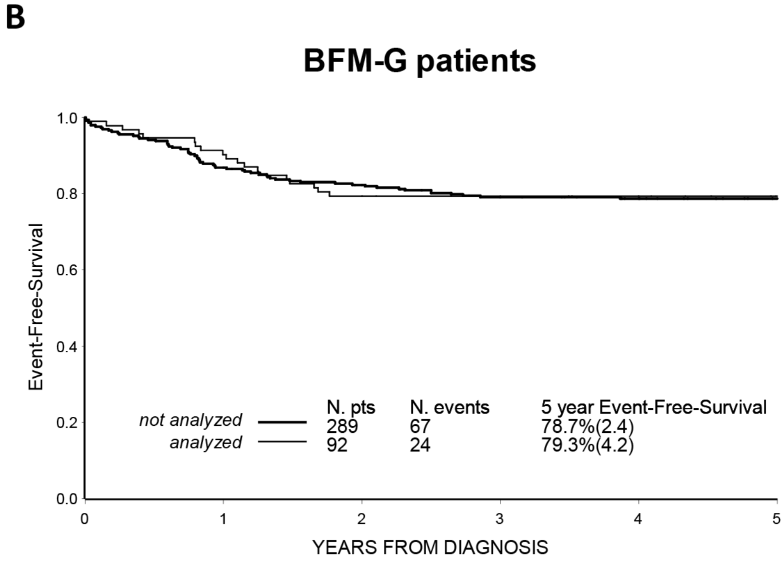

Supplementary Figure S8: Treatment outcome of study cohort. EFS of AIEOP **A.** and BFM-G **B.** patients included and non-included in the study cohort.

Supplementary Table S1: *CRLF2* expression in *CRLF2-high* patients among different high risk features

		CRLF2 expression	
***CRLF2-high* patients**	**N pts**	**Median**	**Range**
**Total**	29		
**Age**			
**1-9 Yrs**	13	9.1	5.2-27.4
**10-17 Yrs**	16	8.8	5.0-81.7
**WBC (X1000/µl)**			
**< 100**	15	9.1	5.0-81.7
**≥ 100**	14	8.5	5.7-27.6
**Prednisone Response**			
**Good**	11	9.2	5.6-27.4
**Poor**	17	8.9	5.0-81.7
**MRD**			
**no-HR**	12	7.4	5.0-27.4
**HR**	5	9.2	8.5-27.7
**Final Risk**			
**no-HR**	10	8.5	5.2-27.4
**HR**	19	9.1	5.0-81.7

WBC, White Blood Cell count; MRD, Minimal Residual Disease; HR, High Risk.

Supplementary Table S2: Molecular features of AIEOP study cohort patients positive or negative for *CRLF2* overexpression

Characteristics	AIEOP					BFM-G				
	P-value	*CRLF2-low*		*CRLF2-high*		P-value	*CRLF2-low*		*CRLF2-high*	
		N	%	N	%		N	%	N	%
**All patients**		103	*100.0*	17	*100.0*		80	*100.0*	12	*100.0*
CRLF2 mutations	-					-				
**No**		71	*68.9*	13	*76.5*		-	-	-	-
**Yes**		0	*0.0*	0	*0.0*		-	-	-	-
**Unknown**		32	*31.1*	4	*23.5*		-	-	-	-
JAK2 mutations	-		*0.0*		*0.0*	-				
**No**		76	*73.8*	14	*82.4*		-	-	-	-
**Yes**		0	*0.0*	0	*0.0*		-	-	-	-
**Unknown**		27	*26.2*	3	*17.6*		-	-	-	-
IL7Rα mutations	0.99					0.18				
**No**		89	*86.4*	13	*76.5*		37	*46.3*	2	*16.7*
**Yes**		5	*4.9*	0	*0.0*		8	*10.0*	2	*16.7*
**Unknown**		9	*8.7*	4	*23.5*		35	*43.8*	8	*66.7*
NOTCH1 mutations	0.75					0.99				*0.0*
**No**		28	*27.2*	5	*29.4*		18	*22.5*	2	*16.7*
**Yes**		42	*40.8*	6	*35.3*		27	*33.8*	2	*16.7*
**Unknown**		33	*32.0*	6	*35.3*		35	*43.8*	8	*66.7*
FBXW7 mutations	0.72					0.99				*0.0*
**No**		59	*57.3*	9	*52.9*		36	*45.0*	4	*33.3*
**Yes**		21	*20.4*	2	*11.8*		9	*11.3*	0	*0.0*
**Unknown**		23	*22.3*	6	*35.3*		35	*43.8*	8	*66.7*
SIL-TAL1	0.46					-				
**No**		82	*79.6*	16	*94.1*		-	-	-	-
**Yes**		16	*15.5*	1	*5.9*		-	-	-	-
**Unknown**		5	*4.9*	0	*0.0*		-	-	-	-

Supplementary Table S3: Clinical features of T-ALL patients enrolled in the Italian and German AIEOP-BFM ALL2000 protocol, analyzed and not analyzed for *CRLF2* expression

Characteristics	AIEOP					BFM-G				
	P-value	Analyzed		Not analyzed		P-value	Analyzed		Not analyzed	
		N	%	N	%		N	%	N	%
**All patients**		120	*100.0*	131	*100.0*		92	*100.0*	289	*100.0*
**Gender**	0.81					0.16				
**Male**		94	*78.3*	101	*77.1*		93	*77.2*	91	*69.6*
**Female**		26	*21.7*	30	*22.9*		27	*22.8*	40	*30.4*
**Age**	0.08					0.51				
**1-5 Yrs**		43	*35.8*	37	*28.2*		31	*26.1*	33	*25.3*
**6-9 Yrs**		26	*21.7*	48	*36.7*		35	*29.4*	29	*22.5*
**10-14 Yrs**		41	*34.2*	36	*27.5*		36	*30.4*	45	*34.2*
**15-17 Yrs**		10	*8.3*	10	*7.6*		17	*14.1*	24	*18.0*
**WBC (X1000/µl)**	0.64					<0,001				
**< 20**		27	*22.5*	35	*26.7*		10	*8.7*	42	*31.8*
**20-100**		41	*34.2*	46	*35.1*		42	*34.8*	50	*38.5*
**≥ 100**		52	*43.3*	50	*38.2*		68	*56.5*	39	*29.7*
**Immunophenotype**	0.07					0.02				
**Early-T**		36	*30.0*	54	*41.2*		18	*15.2*	34	*26.3*
**Thym**		64	*53.3*	50	*38.2*		86	*71.7*	70	*53.3*
**Mature T**		15	*12.5*	18	*13.7*		14	*12.0*	20	*14.9*
**Not specified**		5	*4.2*	9	*6.9*		1	*1.1*	7	*5.5*
**Prednisone Response**	0.47					0.58				
**Good**		77	*64.2*	89	*67.9*		74	*62.0*	83	*63.3*
**Poor**		41	*34.2*	39	*29.8*		44	*36.9*	43	*32.9*
**Unknown**		2	*1.6*	3	*2.3*		1	*1.1*	5	*3.8*
**MRD**	0.33					0.74				
**SR**		16	*13.3*	11	*8.4*		14	*11.9*	17	*12.8*
**MR**		40	*33.3*	53	*40.5*		73	*60.9*	66	*50.2*
**HR**		21	*17.5*	25	*19.1*		20	*16.3*	16	*12.4*
**Unknown**		43	*35.8*	42	*32.0*		13	*10.9*	32	*24.6*
**Final Risk**	0.36					0.71				
**no-HR**		68	*56.7*	76	*58.0*		53	*57.6*	180	*62.3*
**HR**		52	*43.3*	55	*42.0*		51	*42.4*	49	*37.7*

WBC, White Blood Cell count; MRD, Minimal Residual Disease; HR, High Risk; MR, Medium Risk; SR, Standard Risk.
